# The value of treatment choice and clinical prognosis for Riolan’s arch in chronic superior mesenteric artery ischaemic disease

**DOI:** 10.1007/s00384-024-04691-y

**Published:** 2024-07-31

**Authors:** Mengqiang Zhang, Subinuer Maimaitiaili, Run Ji, Chen Tang, Jing Cai, Zhao Liu, Tong Qiao

**Affiliations:** 1 Department of Vascular Surgery, Affiliated Drum Tower Hospital, Medical School of Nanjing University, NO. 321 Zhongshan Road, Gulou District, Nanjing, Jiangsu Province China; 2 Nanjing Drum Tower Hospital, Clinical College of Nanjing University of Chinese Medicine, Nanjing, China

**Keywords:** Riolan’s arch, Superior mesenteric artery, Collateral circulatory vessel, Prognosis

## Abstract

**Objective:**

To explore the value of treatment choice and clinical prognosis for Riolan’s arch in chronic superior mesenteric artery (SMA) ischaemic disease in vascular surgery.

**Methods:**

The clinical data of 215 patients with SMA ischaemic disease (41 cases with Riolan’s arch and 174 cases without) admitted to the Department of Vascular Surgery, Affiliated Drum Tower Hospital, Medical School of Nanjing University (China) from January 2019 to April 2023 were reviewed. Clinical characteristics, imaging findings, treatment, perioperative complications, and patient follow-up data were analysed to observe the impact of Riolan’s arch on the prognosis of patients with SMA ischaemic disease.

**Results:**

There were significant differences in body mass index (Riolan’s arch group: 22.82 ± 3.28 vs 24.03 ± 4.26 in non-Riolan’s arch group, *P* = 0.049), Takayasu’s arteritis (4.9% vs 0, respectively, *P* = 0.036), and secondary intervention (3.3% vs 1.9%, respectively, *P* < 0.001) between the two groups. Propensity score matching was used to exclude the effect of baseline data on patient outcomes. There were significant differences related to therapy method (conservative treatment, Riolan’s arch group: 24.1% vs 39.7% in the non-Riolan’s arch group; operative treatment, Riolan’s arch group: 51.7% vs 20.7% in the non-Riolan’s arch group, *P* = 0.014), as well as in-hospital time (9.79 ± 4.20 vs 6.86 ± 4.32, respectively, *P* = 0.011). There was no statistically significant difference in Kaplan–Meier curves between the two groups (log-rank test *P* = 0.476).

**Conclusions:**

Riolan’s arch plays an important compensatory role in SMA ischaemic disease, especially in chronic disease. We found significant differences in the treatment methods and length of hospital stay of Riolan’s arch, which may suggest that Riolan’s arch has some reference value in the choice of treatment mode.

**Supplementary Information:**

The online version contains supplementary material available at 10.1007/s00384-024-04691-y.

## Introduction

Superior mesenteric artery (SMA) ischaemic disease is a serious life-threatening disease with a high mortality rate and the cause of SMA embolism, SMA thrombosis, non-occlusive bowel SMA ischaemia, SMA dissection, and SMA atherosclerotic stenosis [1]. Disruption of the blood supply to the SMA can lead to intestinal necrosis, which can lead to serious disease and death. The short-term mortality rate of SMA ischaemic disease is high, ranging from 26 to 86% [2]. Therefore, early diagnosis and timely treatment are essential to improve the prognosis of SMA ischaemic disease [3].

Riolan’s arch is a collateral circulatory vessel that is anastomosed by the left branch of the middle colic artery and the ascending branch of the left colic artery. When the SMA or inferior mesenteric artery (IMA) undergoes pathological changes, or the patient has an intestinal tumour, Riolan’s arch can serve as an important vessel for perfusion of the visceral region [4]. With advances in techniques for detecting artery levels, good observation of the condition of the thoracic and abdominal aorta and its branches can be achieved, without the need for traditional autopsies. Riolan’s arch has been reported to fluctuate between 1.9 and 18% [5–9], and it plays an important role in the field of vascular surgery, particularly in cases of regional surgery, where poor decision-making can have disastrous consequences for patients. For example, the presence of Riolan’s arch has important clinical implications for improving the collateral circulation of the colonic vessels; when the SMA or IMA is significantly narrowed or occlusive, the arch is compensatively thickened and tortuously dilated. Approximately 3–7.6% of the Asian population have Riolan numbers [10]. Previous studies on Riolan’s arch mainly focused on case reports and did not carry out systematic investigations. This study aimed to explore the value of treatment choice and clinical prognosis of Riolan’s arch in cases of chronic SMA ischaemic disease in vascular surgery. This was completed via the retrospective analysis of the imaging and clinical data of patients with SMA ischaemic disease and combined with a literature review.

## Methods

### Trial design

This study was designed as a retrospective observational study. This study was approved by the Ethics Committee of the Affiliated Drum Tower Hospital, Medical School of Nanjing University (China) (ethical review no. 2022–556-01). As this was a retrospective study, the need for informed consent was waived. This research was conducted in accordance with the principles of the Helsinki Declaration.

The medical records of 215 patients with SMA ischaemic disease admitted to the Department of Vascular Surgery of the Affiliated Drum Tower Hospital of Nanjing University Medical School from January 2019 to April 2023 were retrospectively 4analysed. Observation indicators included general patient information, such as age, gender, height, body mass index (BMI), preoperative laboratory indicators (ALT, AST, BUN, Cr, Hb, CRP, TG, TC, HDL, LDL), Riolan’s arch and its measurements, perioperative complications, postoperative telephone follow-up, and survival. We used propensity score matching to group patients and analyse the effect of Riolan’s arch on outcomes.

### Inclusion/exclusion criteria

The inclusion criteria were as follows: Patients with computed tomography angiography (CTA) and digital subtraction angiography (DSA) data, or who had undergone open surgery and were diagnosed with SMA ischaemic disease; no age or gender requirements were observed. The observation group included patients with SMA-related diseases combined with Riolan’s arch. The control group included patients with SMA-related conditions who did not have Riolan’s arch.

The exclusion criteria were as follows: (1) SMA aneurysm; (2) gastrointestinal tumour; (3) lack of follow-up data and poor imaging quality; and (4) patients with life-threatening liver and kidney diseases.

### Data acquisition and Riolan’s arch measurements

**Fig. 1:**
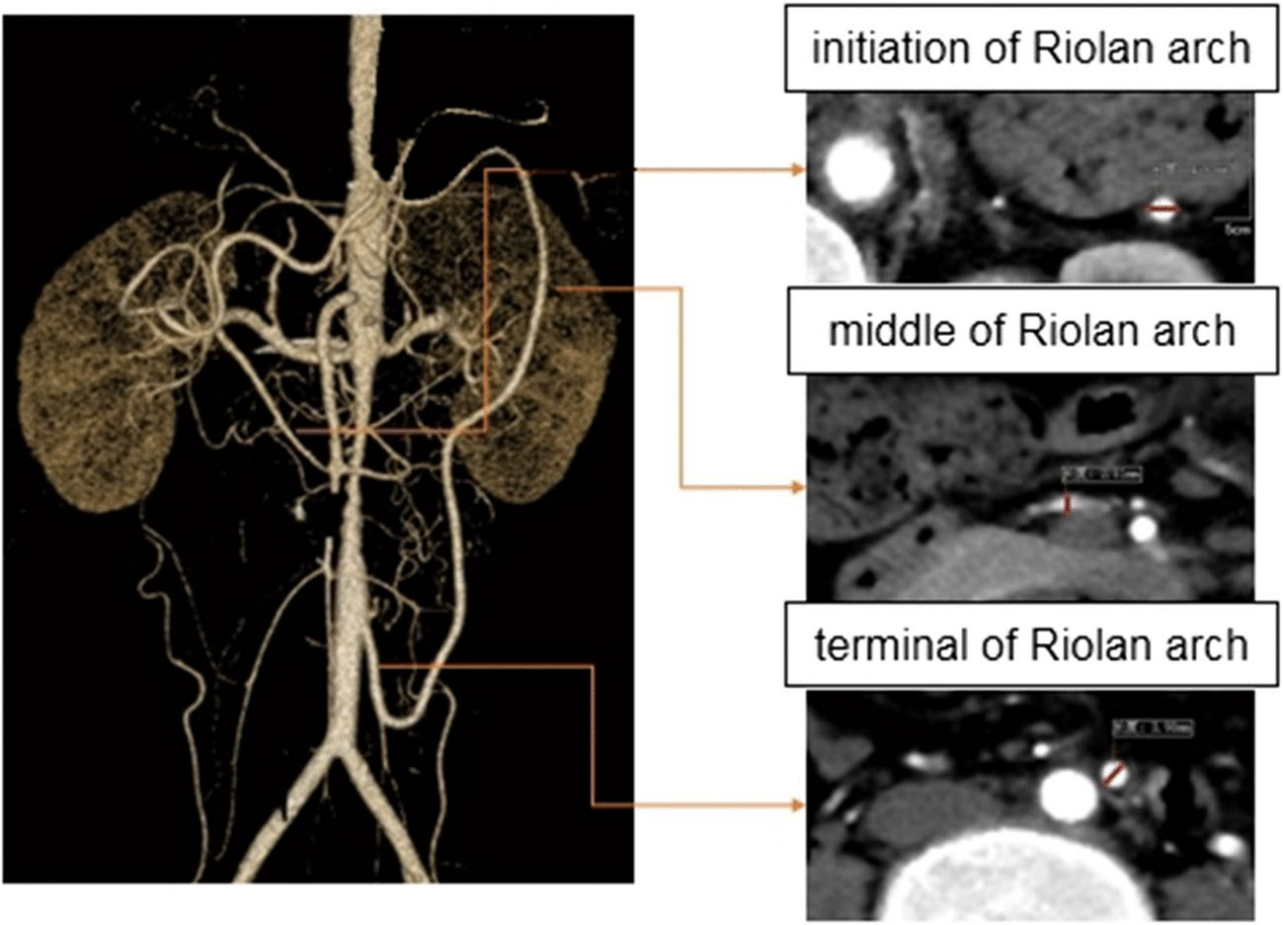
Schematic of the diameter measurement of the Riolan arch

All of the admitted patients underwent chest and abdomen scans using a Siemens Second Generation Flash Dual Source CT (128-row CT). The scope of the scans mainly included the thoracoabdominal aorta and its branches. The CT examination method was as follows: The patient was placed in the supine position and 320 mg/mL of ioxanol contrast agent (iodixanol injection, Jiangsu, China) was injected through a median cubital vein with a double-barrelled high-pressure syringe in preparation for a plain CT and 3-stage dynamic scans (arterial, venous, and delayed stage scan were conducted at 30, 70, and 210 s, respectively). The pitch was 0.914:1 and the thickness of the scanning layer was 5.0 mm with a layer spacing of 1.0–5.0 mm. The following scanning conditions were applied: a flow rate of 4–5 mL/s and a dosage of 1.5 mL/kg, followed by an injection of 20 mL of normal saline. Two radiologists measured the proximal, intermediate, and distal diameters of Riolan’s arch. The Riolan’s arch measurement is shown in Fig. 1.

### Statistical analyses

The SPSS 27.0 software (IBM SPSS Statistics, Armonk, NY, USA) was used to conduct statistical analysis. The measurement data were expressed as mean ± standard deviation, and an independent samples *t*-test was used. Counting data was expressed as examples or percentages using the $\chi$2 test. Propensity score matching was used for grouping and the Kaplan–Meier method was used to conduct survival analysis. The level of significance was set at 5%, and the results were presented with their 95% confidence intervals (CI).

## Results

### Study population

**Fig. 2:**
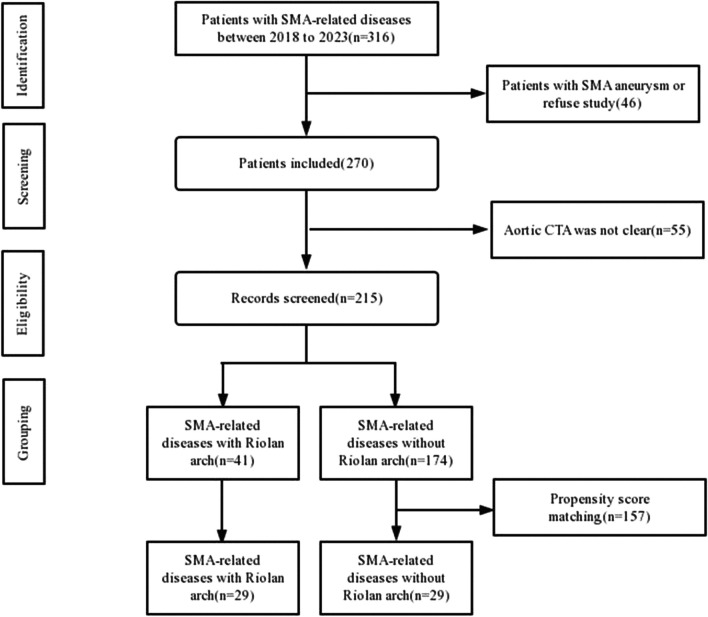
Flow chart of the patient screening. A total of 316 patients with SMA-related diseases were selected and 215 were selected after screening. After propensity score matching, 29 patients were in group Riolan and 29 were in group non-Riolan. SMA, superior mesenteric artery; CTA, computed tomography angiogram

A total of 316 patients with superior mesenteric artery disease were included from 2019 to 2023, of which 46 were excluded due to superior mesenteric aneurysm and 55 patients were excluded due to poor CTA imaging quality. Finally, the clinical data of 215 patients with SMA-related diseases admitted to the Department of Vascular Surgery of the Affiliated Drum Tower Hospital of Nanjing University Medical School from January 2019 to April 2023 were retrospectively analysed. A flow chart showing study population is presented in Fig. 2.

In this study population, 41 patients had a Riolan’s arch, and 175 patients did not. The mean diameter of the arch was 3.54 ± 1.02 mm. There were no significant differences in terms of age, sex ratio, hypertension, diabetes mellitus, atrial fibrillation, therapy method, intestine enterectomy, treatment abandonment, septicopyaemia, intestinal ischaemia, or instances of death between the patients of each group (Table  1), except for BMI (Riolan’s arch group: 22.82 ± 3.28 vs 24.03 ± 4.26 in the non-Riolan’s arch group, *P* = 0.049), Takayasu’s arteritis (4.9% vs 0%, respectively, *P* = 0.036), and secondary intervention (3.3% vs 1.9%, respectively, *P* < 0.001).

**Table 1:** Baseline demographics and characteristics for patients with group Riolan and group non-Riolan

Characteristics	Total (*n* = 215)	Group Riolan (*n* = 41)	Group non-Riolan (*n* = 174)	*t/Z/*$\chi$^*2*^	P*
Sex(male)	159 (74)	27 (65.9)	132 (75.9)	1.726	0.189
Age- y	59.41 ± 12.74	58.63 ± 15.23	59.59 ± 12.12	− 0.432	0.666
Body mass index -kg/m^2^	23.8 ± 4.11	22.82 ± 3.28	24.03 ± 4.26	− 2.000	**0.049**
Diagnosis				25.644	** < 0.001**
SMAD	124 (57.7)	14 (34.1)	110 (63.2)		
SMAO	25 (11.6)	14 (34.1)	11 (6.3)		
SMAS	26 (12.1)	8 (19.5)	18 (10.3)		
SMAT	40 (18.6)	5 (12.2)	35 (20.1)		
Hypertension	109 (50.7)	16 (39.0)	93 (53.4)	2.762	0.97
Diabetes mellitus	22 (10.2)	4 (9.8)	27 (15.5)	0.943	0.332
Atrial fibrillation	31 (14.4)	27 (16.6)	4 (7.7)	3.893	0.345
Takayasu’s arteritis	2 (0.9)	2 (4.9)	0 (0)		**0.036**
CHD	7 (3.3)	0 (0)	7 (4.0)	0.667	0.414
Therapy method				3.721	0.054
Conservative treatment	113 (52.6)	16 (7.4)	97 (45.1)		
Operative treatment	102 (47.4)	25 (11.6)	77 (35.8)		
Surgical method				0.723	0.395
Open operation (*n*, %)	40 (39.2)	8 (7.8)	32 (31.4)		
Interventional operation	62 (60.8)	17 (16.7)	45 (44.1)		
Intestine enterectomy	11 (5.1)	2 (0.9)	9 (4.2)	0	1
Treatment abandoning	7 (3.3)	0 (0)	7 (3.3)		0.351
Septicopyemia	6 (2.8)	0 (0)	6 (2.8)		0.598
Intestinal ischemia	14 (6.5)	3 (1.4)	11 (5.1)		1
Secondary intervention	11 (5.1)	7 (3.3)	4 (1.9)	12.032	** < 0.001**
Death (*n*, %)	15 (7)	3 (1.4)	12 (5.6)	0	1

Data are presented as n (%) or mean ± standard deviation unless stated otherwise

*SMAD* superior mesenteric artery dissection, *SMAO* superior mesenteric artery occlusion, *SMAS* superior mesenteric arteries stenosis, *SMAT* superior mesenteric arterial thrombosis, *CHD* coronary heart disease

^*^P values at *α* = 0.05 were considered statistically significant

Statistical differences were observed between the baseline data of the patients. To eliminate the effect of baseline data on the purpose of the study, we performed bias matching on the data and obtained 29 patients each from the Riolan’s arch and non-Riolan’s arch groups, as shown in Table 2.

**Table 2:** Baseline demographics and characteristics for patients with group Riolan and group non-Riolan after propensity score matching

Characteristics	Total (*n* = 58)	Group Riolan (*n* = 29)	Group non-Riolan (*n* = 29)	t/Z/$\chi$2	P*
Male sex	44 (75.9)	22 (75.9)	22 (75.9)	0	1
Age- y	57.72 ± 12.28	57.21 ± 14.47	58.24 ± 9.84	− 0.318	0.751
Body mass index -kg/m^2^	24.02 ± 3.81	23.88 ± 3.17	24.16 ± 4.4	− 0.275	0.784
Diagnosis				4.004	0.267
SMAD	33 (56.9)	14 (48.3)	19 (65.5)		
SMAO	10 (17.2)	7 (24.1)	3 (10.3)		
SMAS	8 (13.8)	3 (10.3)	5 (17.2)		
SMAT	7 (12.1)	5 (17.2)	2 (6.9)		
Hypertension	23 (39.7)	13 (44.8)	10 (34.5)	0.648	0.421
Diabetes mellitus	4 (6.9)	2 (6.9)	2 (6.9)	0	1
Atrial fibrillation	6 (10.3)	4 (13.8)	2 (6.9)		0.067
ALT	16.35 (10.98–21.35)	16.3 (10.8–20.4)	16.5 (12.5–22.7)	− 0.49	0.624
AST	16.6 (13.48–20.23)	15.3 (13.3–19.2)	17.2 (14.3–20.1)	− 0.723	0.47
BUN	5.04 ± 1.84	5.16 ± 1.85	4.93 ± 1.85	0.478	0.635
Cr	59.5 (53–69.25)	58 (53–76)	62 (53–67)	− 0.257	0.797
TG	1.21 (0.8–1.7)	1.23 (0.78–1.78)	1.19 (0.84–1.5)	− 0.148	0.883
TC	4.22 ± 0.96	4.09 ± 1.13	4.35 ± 0.75	− 1.042	0.303
HDL	1.06 ± 0.35	0.99 ± 0.36	1.12 ± 0.34	− 1.351	0.182
LDL	2.47 ± 0.82	2.37 ± 0.99	2.58 ± 0.6	− 0.956	0.343
CRP	7.85 (3.28–21.38)	14.7 (4.9–34.4)	5.6 (2.5–17.1)	− 2.403	0.016
Hb	133.69 ± 18.85	136.03 ± 19.87	131.34 ± 17.81	0.947	0.348

Data are presented as *n* (%) or mean ± standard deviation unless stated otherwise

^*^P values at *α* = 0.05 were considered statistically significant

To explore the effect of Riolan’s arch on the prognosis of patients, we conducted a correlation analysis of patients’ data in the propensity score matching group. There were no significant differences in terms of the surgical method, intestine enterectomy, treatment abandonment, septicopyaemia, intestinal ischaemia, or instances of death between the patients in the two groups (Table 3); the exceptions here were the therapy method (conservative treatment, Riolan’s arch group: 24.1% vs 39.7% in the non-Riolan’s arch group; operative treatment, Riolan’s arch group: 51.7% vs 20.7% in the non-Riolan’s arch group, *P* = 0.014), and in-hospital time (9.79 ± 4.20 vs 6.86 ± 4.32, respectively, *P* = 0.011).

**Table 3:** The perioperative and follow-up data of patients with propensity score matching

Characteristics	Total (*n* = 58)	Group Riolan (*n* = 29)	Group non-Riolan (*n* = 29)	t/Z/$\chi$2	P*
Therapy method				6.046	**0.014**
Conservative treatment	37 (63.8)	14 (24.1)	23 (39.7)		
Operative treatment	21 (36.2)	15 (51.7)	6 (20.7)		
Surgical method					0.623
Open operation	6 (28.6)	5 (23.8)	1 (4.8)		
Interventional operation	15 (71.4)	10 (47.6)	5 (23.8)		
Intestine enterectomy	2 (3.4)	1 (1.7)	1 (1.7)		1
Treatment abandoning	1 (1.7)	0 (0)	1 (1.7)		1
Septicopyemia	1 (1.7)	0 (0)	1 (1.7)		1
Intestinal ischemia	3 (5.2)	2 (3.4)	1 (1.7)		1
Death	4 (6.9)	2 (3.4)	2 (3.4)		1
In-hospital time-d	8.33 ± 4.47	9.79 ± 4.20	6.86 ± 4.32	2.623	**0.011**
Duration of follow-up-d	711.05 ± 458.58	817.59 ± 504.74	604.52 ± 387.07	1.804	0.077

Data are presented as *n* (%) or mean ± standard deviation unless stated otherwise

^*^P values at *α* = 0 .05 were considered statistically significant

**Fig. 3:**
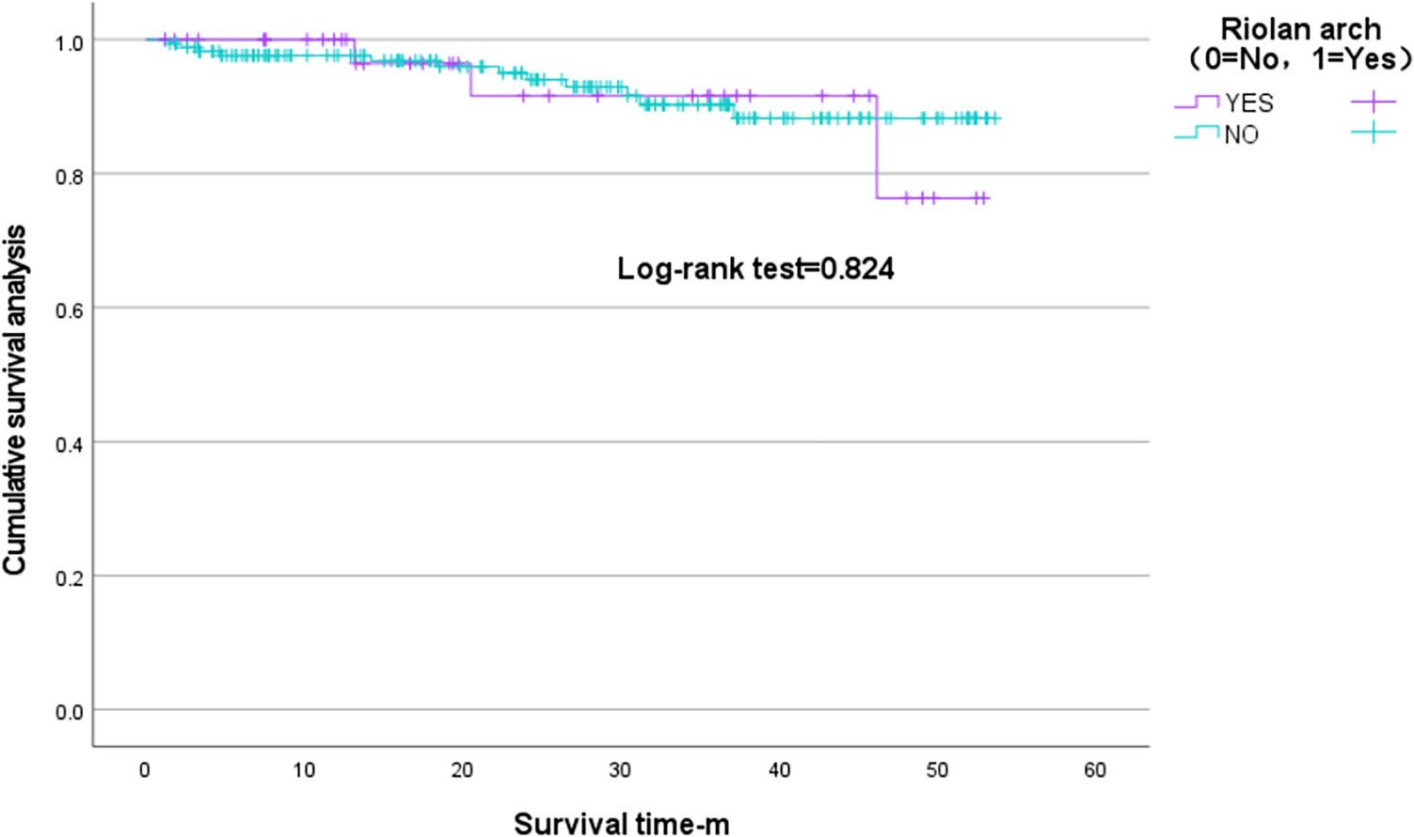
Cumulative Kaplan–Meier estimates for groups Riolan and non-Riolan

No statistically significant differences were observed in the Kaplan–Meier curves for the Riolan’s arch and non-Riolan’s arch groups (log-rank test, *P* = 0.476), with a total of 2/29 events in the Riolan’s arch group and 2/29 in the non-Riolan’s arch group during follow-up (Fig. 3).

## Discussion

In this study, we investigated the important role of Riolan’s arch in ischaemic disease of the SMA. By excluding the interference of baseline data, we found no statistically significant difference in survival between patients with Riolan’s arch and those without (*P* = 0.476). The overall hospital stay of patients with Riolan’s arch was longer (*P* = 0.011), which may be related to the longer visceral ischaemia time and the relatively poor overall status of these patients. This may indicate that Riolan’s arch presents a degree of reference value in the choice of treatment method. Perioperative complications and mortality were not significantly different between the two groups.

Because Riolan’s arch serves as the collateral circulatory vessel between the SMA and the IMA, its occurrence is greatly increased when either the SMA or IMA suffers from atherosclerosis or acute ischaemia, resulting in a corresponding deficiency of blood supply to the bowel [9]. Moreover, the diameter of Riolan’s arch is closely related to the type of disease and the length that ischaemia has been present. Xie et al. reported Riolan’s arch diameters of 4.6 and 2.5 mm, respectively, when the SMA was blocked and narrowed [5]. The mean diameter of Riolan’s arch in our study was 3.54 ± 1.01 mm.

Based on our findings, there were no statistically significant differences between the two study groups in terms of surgery method, enterectomy, abandonment of treatment, sepsis, intestinal ischaemia, or death, but there were statistically significant differences in terms of treatment and length of hospital stay. There are currently few reports on the incidence of these indicators in the presence of Riolan’s arch in chronic SMA ischaemic disease.

The difference in treatment between the two groups in our study may have resulted for several reasons. For example, as the duration of the stenosis lengthens, the diameter of Riolan’s arch may increase compensatively and relieve clinical symptoms. If Riolan’s arch delivers poor compensation, patients may experience post-meal abdominal pain, weight loss, and even intermittent claudication [11, 12]. Alternatively, when the compensation is satisfactory, patients can avoid surgery. Second, there was a significant difference in the length of hospital stay between the two groups. This further suggests the prognostic value of Riolan’s arches in the treatment of SMA chronic ischaemic disease. However, this result requires further confirmation and support from future studies.

**Fig. 4:**
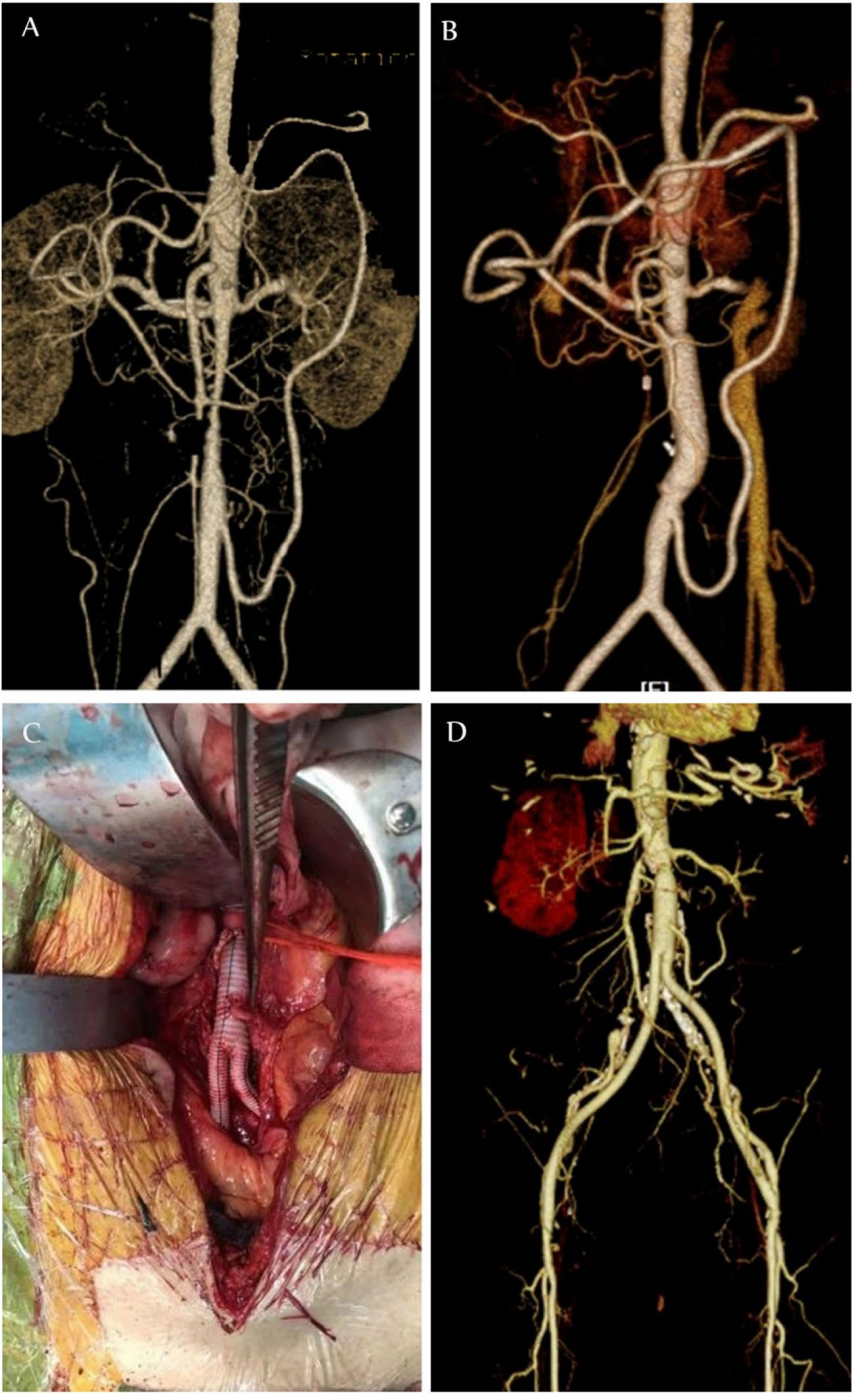
**A** Riolan arch in patients with abdominal aortic stenosis. **B** IMA is protected during abdominal aortic stent implantation. **C** IMA was anastomosed to artificial vessels. **D** CTA after abdominal aortic resection with artificial vessel replacement. IMA, inferior mesenteric artery; CTA, computed tomography angiogram

The clinical situation is complex, and the causes leading to the formation of Riolan’s arches vary. Some patients have superior mesenteric artery occlusion, resulting in a very developed inferior mesenteric artery and the formation of Riolan’s arch, while some patients have inferior mesenteric artery occlusion and the superior mesenteric artery is more developed and the formation of Riolan’s arch. Reasonable and effective surgical methods must be developed for different situations. Patients with abdominal aortic stenosis combined with SMA occlusion tended to have rheumatic conditions such as aortitis [13]. These patients are often combined with Riolan’s arches, and when performing surgical treatments (whether open or interventional), the protection of Riolan’s arches must be considered (Fig. 4). During the procedure, the IMA can be anesthetised with artificial blood vessels or stents that do not affect the blood supply to the IMA, thus ensuring blood supply to the visceral arteries and avoiding serious complications such as intestinal ischaemia or even intestinal necrosis.

Riolan’s arches have been linked to clinically relevant complications. Type II endoleak after endovascular isolation of abdominal aortic aneurysm may be caused by collateral artery countercurrent, which may be related to the crossing of Riolan’s arch through the inferior mesenteric artery. According to the literature, the incidence of a type II endoleak is approximately 10–44% [14]. Guidelines recommend intervention for a type II endoleak in an aneurysm body larger than 10 mm [15]. If the IMA is a criminal vessel, embolisation can be performed via Riolan’s arch.

It is well known that the IMA is typically sacrificed during endovascular aortic repair (EVAR) therapy for abdominal aortic aneurysm (AAA); however, it must be retained in exceptional cases. When AAA is combined with SMA stenosis or occlusion, the Riolan’s arch is usually present. At this point, the IMA should be retained during surgical treatment; otherwise, it could lead to serious complications, such as internal ischaemia and even intestinal necrosis in the patient [16].

The Riolan’s arch also plays an important role in isolated SMA dissection (ISMAD), which can be broadly classified into 3 types based on the Yun classification. The higher the classification, the more severely the intestinal tract is affected by ischaemia, and the more developed the Riolan’s arch [17]. Huang et al. found that when patients undergoing ISMAD had a Riolan’s arch on admission, their pain scores were lower, and the time they required relief was shorter. Riolan’s arches may alleviate visceral ischaemia to some extent in acute conditions, thus buying time for patients to undergo surgery and improving their prognosis [16].

To the best of our knowledge, this is a very important study of the Riolan's arch so far, which has, until now, primarily been recounted in case reports. Based on this study, we discovered the following. (1) It is possible that compensation of the vascular arch may alleviate symptoms and delay treatment of the mesenteric artery related diseases or delay the duration of treatment. (2) There is a focus on protecting the vascular arch during treatment and avoiding acute ischaemia. (3) Evaluation of the vascular arch in certain diseases can help to predict the effectiveness of surgery or the need for reconstructing the arch.

## Study limitations

This research has several limitations. (1) The study was a retrospective, single-centre study, and biases were present in the collection of patient information. (2) The small sample size of patients may have affected the results, making them less reliable. (3) Riolan’s arch measurements are subject to human error, which can affect research results. (4) Follow-up was conducted primarily via telephone, and an understanding of the patient’s true condition may not have been accurate, which may affect subsequent outcomes. (5) By reviewing only the medical records when collecting data, the true condition of patients may not have been accurately recorded.

## Conclusions

This is the first study to use Riolan’s arch as a variable for studying the clinical outcomes in patients where the arch plays an important role as a collateral circulatory vessel. Longer hospital stays for Riolan’s arch patients suggest that patients with poorer underlying conditions may need to be hospitalised for extended periods. The long-term survival rate was optimistic in patients with Riolan arch, but there were no statistically significant differences. Larger study populations are needed, as well as more rigorous trial protocol designs for exploring the effects of Riolan’s arch on patients.

## Supplementary Information

Below is the link to the electronic supplementary material.

Supplementary file1 (DOC 100 KB)

## Data Availability

All data generated or analysed during this study are included in this published article.
